# The Horse’s Mouth2Read at the November meeting of the Veterinary Medical Association of New York County.

**Published:** 1898-12

**Authors:** Frederic C. Grenside

**Affiliations:** New York City


### THE HORSE'S MOUTH.[2]

By Frederic C. Grenside, V.S.
NEW YORK CITY.

Under this somewhat ambiguous title I do not purpose discussing the ordinary diseases of the mouth so fully treated of in textbooks, but rather wish to direct your attention to this important organ of the horse, and view it to some extent from the practical horseman's standpoint.

It may be asked, why introduce a subject that comes within the province of the practical horseman at a meeting of veterinarians? In reply, I may say I think I can show that when the mouth is studied from the standpoint of an organ by which the horse is controlled and guided, conditions arise which cause the provinces of the practical horseman and veterinarian to overlap, and it would puzzle a constitutional lawyer to strike the dividing line.

While it is not absolutely necessary for a veterinarian to be a practical horseman under all circumstances (though it always is an advantage), conditions arise referable to the mouth in which it is a great assistance in aiding one to explain faults and troubles to our clients to which driving and riding horses are subject, and to suggest means for their relief.

[2] Read at the November meeting of the Veterinary Medical Association of New York County.

I think we will all agree that there is no point in connection with a horse that contributes so much to the pleasure, comfort, and safety of either riding or driving him as what might be called a responsive mouth, or one which obeys the slightest intimation promptly, from rider or driver, of restraint or guidance. A good mouth is to a large extent natural to a horse, so that some horses if properly handled can have their mouths made almost perfect. Such horses, as a rule, must have their heads so related to one another that they can bend their heads upon their necks with ease. If horses so formed have bad mouths it is usually the result of irrational handling, unless they happen to be unduly nervous or unintelligent animals.

Horses whose mouths are not good are very subject to soreness occasioned by injury from the bit, and the result of this soreness is manifested in a variety of ways.

In horses driven with curb-bits with a stiff mouthpiece the usual seat of injury is the tissue covering the branches of the lower jaw, at the points where the bit presses, which become bruised and excoriated, the bone underlying being sometimes injured even to the extent of a piece being chipped off. It is extraordinary how common this form of injury is in the city, especially amongst dealers' horses, and is by no means uncommon in a large proportion of other horses driven with curb-bits. Jointed or snaffle-bits seldom injure the branches of the lower jaw, but sometimes press the cheeks against the anterior molars, and abrade the inner surface of the cheeks, especially if these molars are rough.

Of the numerous ill-results of soreness and discomfort in connection with the mouth I may mention the following faults and troubles noticeable when riding or driving, viz.: crossing the jaws keeping the mouth more or less open, lolling the tongue, slobbering, tossing the head, carrying the head to one side or the other, pulling out in double harness or crowding in, going cornerwise, side-lining, not going into the bit, carrying the head unsteadily, pulling, boring down, balking, rearing, plunging, or rushing when starting off, especially out of the stable, restlessness in standing, breaking or going unsteadily in harness when going within the horse's speed, mixing, hitching or hopping either in front or behind, interfering, and last, but not least in importance, bridle-lameness. Certainly a number of other causes operate in producing the faults I have enumerated, but the most prolific one in the majority of instances is some discomfort in connection with the mouth.

As a rule, if these troubles are attributed to the mouth by owners or coachman, the teeth are usually assigned as the cause, when in reality a bruise of the jaw, occasioned by the bit, is the trouble; but the anterior molars are rasped and rerasped, still the source of irritation (the bit) is used day after day, applied to the sore and tender spots.

If one considers for a moment one can realize the extreme sensitiveness of these sores, and the excruciating pain a horse must suffer when facing the bit in the morning, so that it is not astonishing that some horses hang back when first taken out, and if they are predisposed become balkers. The high-couraged horse, though he may hesitate at first, will, as soon as the part becomes numbed with pressure, or he becomes desperate with the pain he is suffering, begin to pull and show evidence of the discomfort he is suffering in the many ways already described, such as crossing the jaws, going with the mouth open, head to one side, etc. The irritable, sensitive horse is apt to manifest his pain in a more demonstrative manner, and we may find him going out of the stable with a rush, rear, or plunge, and if he continues to do this for a short time it soon becomes a confirmed habit and a very dangerous and disagreeable one.

Unfortunately, the condition is by no means uncommon, and could be easily prevented were it realized that it is due to soreness of the mouth, and rational measures adopted. Instead, however, of resting the mouth, by keeping the bit out of it, the horse is used day after day and the condition aggravated. If the excoriated parts heal, the cicatricial tissue filling the breach gives rise to an uneven surface, and the healed part remains unduly sensitive, and the pressure of the bit always causes evidence of discomfort. Some refer to the healed part as calloused and lacking in sensitiveness, and so account for some horses having a one-sided mouth, but personally I think it is the healed part that is the sensitive one.

Just here I may remark that I think this is a point that should not be ignored by the veterinarian in examining for soundenss, and should at least be pointed out to his client, and its consequences explained; the more so, if any injury to the bone has ever taken place, for then a horse can never have a good mouth.

Outside of the discomfort and difficulty of driving a horse with a bad mouth, not to say the danger, especially in crowded streets, and the unsightliness of carriage it gives rise to, as in turning the head in and out, etc., a bad mouth is apt to produce irregularity of the gait and impaired control of the legs. What is called "hitching" or hopping off one leg, generally a hind one, although due to weakness, too heavy a load, driving beyond speed, heavy shoes, etc., is not infrequently due to tenderness or soreness of the mouth, or placing the bit too high in the mouth. There is no such thing as a congenital "hitcher." It is always the result of bad management if allowed to become a habit. In high-couraged horses, whose mouths have become permanently injured from the bit, it is a difficult matter to overcome the habit; but if the mouth is allowed to heal thoroughly, the bit placed as low in it as the animal will stand and face it with a moderate degree of firmness, and not put his tongue over it, the fault will often be remedied.

The veterinarian needs to be on the alert for seeming lameness from a sore mouth, which is by no means uncommon. A horse will nod his head or hitch on a hind leg as rhythmically as if he were actually lame, and owners and coachmen often jump at the conclusion that such is the case, neglecting to take the precaution to jog a horse in hand before coming to the conclusion. In fact, it is very difficult to persuade people sometimes that a horse is not lame when he nods or hitches from a sore mouth. I have known horses to go apparently lame when driven with a certain kind of bit, that would go naturally with another kind, and I have also known horses to go seemingly lame when driven on one side in a pair, that would show nothing irregular when driven on the other side. One is more apt to have an experience of this kind with green horses that are being trained to a curb-bit than with those that are seasoned, unless their mouths have been permanently injured, and those driven in double harness are more apt to show it than those used in single harness.

In standing about show- and sale-rings one frequently hears horses condemned as being lame when it is due to soreness of the mouth, the tendency to which is increased by going around a small ring.

Mixing is usually attributed to want of balance from a proper distribution of weight in shoeing, and no doubt this is the case in some instances, but I think the cause should be more frequently referred to the mouth. You will generally find a horse that is inclined to mix has an unsteady mouth. He does not take the bit with the necessary firmness, and keeps retracting his tongue or putting his tongue over the bit so that the pressure from the bit comes on the branches of the lower jaw, which always gives rise to irritability and a want of confidence in the animal's manner of going.

The tendency to mix can usually be overcome by patient and persevering effort to get the tongue accustomed to pressure. The bit should be placed well up in the mouth and be as comfortable a one as possible; sometimes a bit with a flexible-rubber mouthpiece or an arched stiff one; leaving the bit in the mouth in the stable for several hours daily, so as to get the tongue used to its pressure, so that it will remain quietly under a bit; apply gradually increasing pressure from day to day by means of a dumb jockey. Sometimes a bit with a post will remedy the trouble at once; but, as a rule, the former plan is the best.

Carelessness in the position in which the bit is placed in the mouth often results in injury to that organ. It is a point that a driver should exercise as much vigilance about, with almost as much care, as determining whether the reins are buckled to the bit. The lower the bit is placed in the mouth, within certain limits, the better, providing the horse will take it. With a moderate degree of firmness keep his head steady and his tongue under it. In those horses, however, which do not force the bit steadily, it is usually better to raise it in the mouth, and as the mouth becomes firmer lower it.

Among the exciting causes of "interfering," soreness of the mouth is by no means an uncommon one, and I have frequently observed it occurring even in well-broken horses, when a change of bit, particularly to a severe one, had produced some injury to the mouth. Fatigue, bad shoeing, rough and slippery roads, the swaying of a heavy two-wheeler are all exciting causes of "striking" or "brushing," but I am of the opinion that the awkwardness arising from an imperfectly made (not thoroughly bitted) mouth, with the incidental soreness, is an important factor.

